# Cross-species comparisons and *in vitro* models to study tempo in development and homeostasis

**DOI:** 10.1098/rsfs.2020.0069

**Published:** 2021-04-16

**Authors:** Teresa Rayon, James Briscoe

**Affiliations:** The Francis Crick Institute, London NW1 1AT, UK

**Keywords:** tempo, stem cells, homeostasis, developmental timing, allochrony, developmental biology

## Abstract

Time is inherent to biological processes. It determines the order of events and the speed at which they take place. However, we still need to refine approaches to measure the course of time in biological systems and understand what controls the pace of development. Here, we argue that the comparison of biological processes across species provides molecular insight into the timekeeping mechanisms in biology. We discuss recent findings and the open questions in the field and highlight the use of *in vitro* systems as tools to investigate cell-autonomous control as well as the coordination of temporal mechanisms within tissues. Further, we discuss the relevance of studying tempo for tissue transplantation, homeostasis and lifespan.

## 1. Introduction

Biological processes comprise ordered events unfolding at characteristic speeds. This is particularly obvious during embryonic development where the sequence and rate of events ensure that structures develop in the right place at the right time. Importantly, the speed of these processes (tempo) controls the rate of development of the whole organism and ultimately the length of time embryogenesis takes. Modifying the pace of development can affect the final size and composition of tissues in a growing organism. Imbalances in the speed of tissue development and stem cell differentiation can result in tissue overgrowth or deficits. Therefore, controlling the speed of differentiation is essential for the size, function and shape of developing organisms.

Biological clocks need to keep track of multiple timescales, and cells need to be in synchrony to build tissues and organs during development and homeostasis. How tempo is encoded in the genome and how organisms coordinate the development of different organs remain unknown. Growing evidence suggests that tempo can be set by autonomous mechanisms in individual cells during development [1,2], but little is known in adult tissues. Likewise, local and global tempo must be coordinated for appropriate developmental outcomes and during homeostasis in the adult. Intra-organ and inter-organ communication mechanisms have started to be deciphered in the regulation of developmental growth and the homeostatic maintenance of tissues [3–5].

Gene regulatory networks (GRNs) play a central role in development. Gene expression programmes are controlled by transcriptional regulators that, together with the cis-regulatory elements to which they bind, integrate external cues and coordinate the spatial and temporal elaboration of developmental programmes [6]. Experimentally, GRNs have been described based on gain and loss-of-function studies of transcription factors and the identification of cis-regulatory elements that drive tissue-specific gene expression profiles. However, most of the methodologies to study transcription factor function have a poor temporal resolution, making it difficult to understand GRNs quantitatively and dynamically. This is starting to change. Recent efforts are beginning to measure mRNA and protein levels and their turnover to incorporate dynamic interactions that are not obvious from static assays [7]. Nevertheless, quantitative and dynamic information on regulatory interactions through cis-regulatory elements is still lagging. *In silico* models and simulations from experimental data using dynamical systems theory can be used to investigate how GRNs dynamically work [8]. Moreover, recent advances in computational inference based on large-scale transcriptome sequencing provide new approaches to gain a systemic understanding of GRNs [9]. Therefore, the combination of new experimental approaches and dynamic modelling provides a framework to investigate the mechanistic basis of developmental tempo.

A fascinating but complex aspect of temporal control is how different developmental processes are regulated at different scales [10]. Transcription factors bind DNA and initiate transcription at timescales of seconds. Within minutes, proteins are produced and activate target genes that create states that persist from minutes to hours. In many cases, the changes in gene expression patterns that characterize developmental stages and result in cell fate choices occur over hours or days, and the whole process of embryo development takes weeks or months in mammals. Techniques with sufficient temporal resolution are essential to gain an understanding across timescales.

One promising approach is to take advantage of the evolutionary conservation of developmental mechanisms and GRNs. Highly conserved GRNs that operate at different speeds in different species permits a search for the properties that might explain differences in tempo. Conserved GRNs are composed of the same regulatory interactions and the same transcription factors. Therefore, the dynamics of molecular processes (transcription factor binding affinities, mechanisms of mRNA and protein production and degradation, RNA splicing, etc.) can be measured and parametrized using dynamical system models to investigate temporal aspects in the GRN. This provides a means to explore how conserved GRNs operate proportionally at different speeds and what the dynamic limits might be. Overall, measurements and perturbations of conserved GRN kinetics combined with *in silico* models will offer insight into how developmental processes generate evolutionary scalable and robust temporal patterns.

## 2. Tempo at the phylotypic stage

During development, the phylotypic stage corresponds to the period of development when embryos manifest the archetype of the vertebrate body plan and hence different species closely resemble one another [11]. This stage comprises the events responsible for the generation of the major tissues of the body prior to the elaboration of species-specific morphological traits that are evident at the completion of organogenesis.

The rate at which embryos from different species progress through the phylotypic stage is species-specific. Since each species develops at a different pace, comparisons between developmental processes at the phylotypic stage are an attractive system to study tempo control. Recently, two papers have taken a comparative approach across species in developmental processes that take place at the phylotypic stage to study tempo: somitogenesis and motor neuron formation in mouse and human embryos [12].

**Figure 1.**
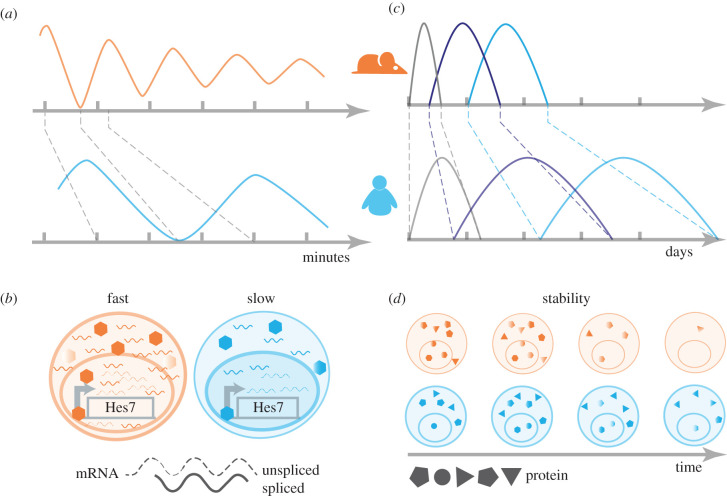
Cell-autonomous tempo at the phylotypic stage. *In vitro* models of the segmentation clock (*a*,*b*) and motor neuron differentiation (*c*,*d*) show that the mouse (orange) processes advance faster than in human (blue). In the segmentation clock (*a*,*b*), the period of Hes7 oscillation serves as a model to measure the difference in tempo. This is due to biochemical reaction parameters (dynamics of transcription and translation) being slower in human compared to mouse. During motor neuron differentiation (*c*,*d*), the temporal progression of gene expression is proportionately slower in human compared to mouse. The differences in progression in motor neuron differentiation correlate with an increased stability in human proteins compared to mouse. Motor neuron model adapted with permission from *Science* (License Number 5002560107532).

One process characteristic of the phylotypic stage in vertebrates is somitogenesis—the process by which mesodermal cells rhythmically produce pairs of segments along the axis that will give rise to muscle and axial skeleton [13]. This involves the periodic oscillation of a GRN known as the segmentation clock. The segmentation clock shows tempo differences across vertebrate embryos. Oscillations of Hes7, the principal transcriptional regulator of the process, last around 30 min in zebrafish, 2–3 h in mouse and 5–6 h in human [14,15]. Recent work has shown that the interspecies period difference is mirrored *in vitro* in tissue derived from the directed differentiation of pluripotent stem cells [16–18]. Since *in vitro* models of the segmentation clock from mouse and human retain the differences in oscillation periods, they can be used to interrogate what determines these differences in the oscillation period. In an effort to understand the mechanism that explains the differences in tempo, Matsuda *et al*. [19] showed that swapping the human Hes7 genomic locus for the mouse locus in mouse embryos does not influence the oscillation period. Instead, they found that the interspecies period difference depends on the kinetics of Hes7. A mathematical model fitted to experimental measurements of degradation rates and delays of HES7 protein and mRNA supported this hypothesis. This demonstrated that the slower kinetic properties of the delayed negative feedback loop of HES7 in human compared to the mouse is sufficient to explain the tempo differences between species [19] (figure 1).

The spinal cord also develops during the phylotypic period and undergoes a series of molecular and morphogenetic processes resulting in the formation of the range of neuronal cell types found in the adult [20]. The specification of one of these neuronal subtypes, motor neurons, is a prominent example of tempo differences in development. Motor neuron differentiation lasts less than a day in zebrafish, 3–4 days in mouse and around 2 weeks in human [21]. To identify the molecular differences that explain differences in pace, we looked in detail at the pace of differentiation of motor neurons in human and mouse. By differentiating motor neurons from mouse and human stem cells, we found a temporal scaling factor of around 2.5 between mouse and human. That is, each stage of motor neuron differentiation took approximately 2.5 times longer in human than in mouse. Neither differences in sensitivity to extrinsic signals nor differences in DNA sequence of key genes could explain the differences in tempo. Instead, a slower temporal progression in human corresponded to increased protein stability. This indicated that changes in protein stability across species may explain differences in tempo [22] (figure 1). Thus, both somitogenesis and motor neuron differentiation showed a proportional temporal scaling factor of 2–3 between mouse and human, which we termed allochrony, as a type of heterochrony. In contrast with the broad meaning of heterochrony, which includes the disproportionate change in timescales of the same process as well as the shift in the time of initiation of a process, allochrony refers to proportionally scaled changes in the pace of development across species.

These two studies on allochrony present some apparent discrepancies. For instance, differences in mRNA stability had an effect on the segmentation clock period between mouse and human, whereas there was no obvious difference in transcript stability during motor neuron differentiation. This could be explained by the relevance of mRNA kinetics with respect to the timescale of the process. The total duration of the segmentation clock is 2–3 h in mouse, whereas motor neuron differentiation lasts over 3 days. Given that mRNA turnover occurs in the range of minutes, changes in mRNA dynamics are predicted to have a larger effect on a process that takes place over 2 h compared to one that takes 3 days. Consistent with this, computational simulations of a mathematical model of the motor neuron GRN showed that a fourfold difference in mRNA stability would be required to accommodate the 2–3 temporal scaling factor measured between mouse and human. By contrast, a twofold change in protein stability would be sufficient to explain the allochrony of the system in the model [22].

Another difference between the reports is that while many genes in the motor neuron transcriptional programme seemed to follow the temporal scaling characteristic of the overall difference in timescale between mouse and human, not all genes in the presomitic mesoderm showed interspecies differences. Differences in the experimental methods used to quantify protein stability could explain this discrepancy. While Matsuda *et al*. [19] overexpressed the reporter proteins of interest and measured luciferase activity to estimate protein half-lives, Rayon *et al*. [22] performed metabolic labelling combined with a pulse and chase strategy to estimate the decay rate of endogenous proteins. Given that both studies measured the protein stability of only a handful of proteins, it will be interesting to systematically compare endogenous protein stability genome wide to characterize and compare the mechanisms further.

In sum, *in vitro* comparisons in mouse and human of the segmentation clock and motor neuron differentiation identified mechanisms that could account for species differences in tempo. Moreover, the preservation of species-specific tempo *in vitro* supports the idea that global temporal scaling mechanisms arise from cell-autonomous processes. Perturbations of developmental progression using *in vitro* systems will now be needed to investigate the role of these mechanisms further. An advantage of *in vitro* systems for this is that they allow controlled manipulations of signalling molecules, morphogen gradients and cell–cell interactions within a population of cells [2–4]. Interspecies co-culture of stem cells might be sufficient to affect components of the tempo in cells from another species *in vitro* [23]. It will be interesting to investigate which are the factors that extrinsically regulate tempo from another species and how they operate. Likewise, it would be interesting to investigate tempo in endoderm derivatives to generalize the allochrony at the phylotypic stage across germ layers.

## 3. Regulating and synchronizing developmental tempo

Complementary to the autonomous control of tempo observed using *in vitro* assays, evidence points to mechanisms that synchronize clocks within and between different tissues. A well-established example is the compensatory growth of early mouse embryos with reduced cell numbers [24]. In this model, mitomycin C, a chemical that inhibits DNA synthesis and cell proliferation, was used to induce cell death between E6.5 and E7.0 during mouse gastrulation. This reduced cell number in the epiblast to around 75% of the normal cell number. During the subsequent compensatory growth, somite differentiation was delayed as somites started to form at E8. From E10.5 onwards, somitogenesis accelerated resulting in the correct somite number by E11.5 [25–27]. Moreover, the formation of head folds was initially delayed, but neural development was then restored to the normal schedule soon after [28]. Together, these results indicate that tissues adjust their cell-autonomous tempo independently, but that there is concerted coordination of temporal progression to achieve the right shape and size by the end of the phylotypic stage, at E12.5. The mechanism behind the coordination of tempo to allow compensatory growth is yet to be determined.

The recent development of three-dimensional stem cell differentiation models, termed gastruloids, offers new possibilities to compare developmental progression and coordination between tissues in mouse and human [29]. These *in vitro* systems break symmetry, grow axially and differentiate cells of all three germ layers [30–32], enabling the comparison of developmental progression between tissues and across species. Benchmarking of gastruloids to mouse and human embryos shows the equivalence between *in vitro* and the *in vivo* timing. Mouse gastruloids closely resemble the developing mouse embryo from E6.5 to E9.5 [33]. The temporal profiling of human gastruloids, in their various versions, suggests that *in vitro* gastruloids take between 3 to 10 days to recapitulate a process that lasts around a week *in vivo*: from CS6 (day 14) to CS9 (day 18–21) [30,31]. The mouse and human timescales of gastruloids suggest that the allochrony of the system may be conserved. It is also worth noting that despite their name, the developmental processes that take place in gastruloids closely resemble those occurring around the phylotypic stage, post-gastrulation. It will be interesting to measure, perturb and compare tempo across species in gastruloids.

Xenopus animal caps and explants of whole zebrafish embryos, termed pescoids, have been shown to maintain the same self-organizing properties as mammalian cells [34]. Comparing temporal progression across these models in mammal and anamniote embryos will extend the study of allochrony across vertebrates. Together, three-dimensional models of development offer a great platform that will allow the study of the coordination of tempo across tissues essential in the formation of the vertebrate body plan. In the future, exploiting compensatory growth experiments in gastruloids may provide clues about the mechanisms of coordination.

## 4. Tempo outside the phylotypic stage

The phylotypic stage arises as the most constrained period of development, requiring the coordination of molecular and morphological processes in the entire embryo [11]. As a consequence, earlier and later processes in development should have a higher degree of variation allowing for species-specific differences. This has been termed the ‘hourglass' model of development [35]. Comparative studies analysing whole embryos or organs at various developmental stages generally support this idea [36–38]. However, multiple differentiation processes take place at the phylotypic stage, and whether temporal progression of these is more conserved in the phylotypic stage than at early and late stages remains to be determined. Higher resolution studies are needed to quantitatively investigate the temporal progression in the formation of specific cell types. Nevertheless, since allochrony has been identified at the phylotypic stage, it is worth asking if processes outside the phylotypic stage show proportional differences in tempo (allochronies) and whether there is a higher degree of variability in the initiation or shifts in temporal progression (heterochronies). We will discuss developmental tempo outside the phylotypic stage looking at comparisons between species where time can be tracked unequivocally.

## 5. Cleavage stage embryos

Upon fertilization, the single-cell zygote undergoes multiple rounds of cell divisions without an increase in mass (this is termed cleavage) and forms a ball of cells called the blastula. Embryos at this stage can self-organize without the need for external cues and can be grown *ex vivo* for long periods of time (up to 4 days in mouse) [39]. Moreover, these are the only accessible stages in human development that are amenable to whole embryo experiments.

Pre-implantation development follows similar morphological states across mammals and results in the formation of a fluid-filled blastocyst. Several perturbation experiments suggest a cell intrinsic control of tempo. Removal of one blastomere from the two-cell embryo in mouse embryos, halving the size of the embryo, leads to the formation of a smaller blastocyst that cavitates at the same time as full embryos [40].

Likewise, dissociated cells from the eight-cell embryo temporally progress at the same rate as intact embryos [41]. However, there are differences between species at these developmental stages that may indicate a lack of GRN conservation and could complicate the comparison of cleavage stages across species. For example, the timing of embryonic genome activation differs, occurring in the 2-cell stage in rodents, between the 4- and the 8-cell stage in humans, and at the 8- to 16-cell stage in sheep and cattle [39]. In addition, the restricted expression of lineage-specific factors occurs much earlier in mouse than in other mammals and might be explained by species-specific enhancer usage [39].

Despite the divergence in genome activation and gene expression patterns, the morphological changes during pre-implantation development are remarkably conserved across species. These morphological changes correlate with the timing of other developmental events such as lineage specification and progress cell autonomously. Morphokinetic analysis using time-lapse imaging in mouse and human embryos from the eight-cell stage up to the blastocyst stage allows for an accurate comparison of developmental progression. The analysis shows that mouse embryos take 12 h from the time of compaction to the formation of an early blastocyst, with a single dominant cavity, whereas it takes 24 h (a twofold difference in tempo) for the same process to occur in human embryos [42].

The twofold tempo difference in morphological changes between mouse and human raises the possibility of allochrony during pre-implantation development. The conservation of allochrony for morphological changes in the system despite the divergence in the GRNs might indicate that there are mechanisms that preserve tempo throughout development. It would be interesting to determine whether protein turnover during pre-implantation development is slower in human compared to the mouse as described for the phylotypic stage.

## 6. Limb morphogenesis

Limbs represent one of the best-studied systems in evolution and development. Although they show a well-defined sequence of temporal events, such as the proximo-distal patterning of the limb bud and the chondrification of skeletal elements, they have undergone extensive evolutionary diversification in different species [43]. Some of this diversification appears to depend on changes in timing [44]. Limbs develop from the flanks of the trunk into three proximo-distal main segments in a proximo-distal timely manner. Cell extrinsic and intrinsic timing mechanisms in the limb operate sequentially and determine the timing of patterning along the antero-posterior axis and the outgrowth termination of the limb bud [45–47].

As limb development proceeds, paired limbs develop to attain a characteristic morphology that ultimately defines the identity of a forelimb (arm) or a hindlimb (leg). The earlier stages in limb development take place during the phylotypic stage, and fore- and hindlimb expression patterns diverge at later stages of development [48]. The disproportionate change in the growth rate of in the fore- versus hindlimbs at later stages allows for the variability in shapes and forms [49], and shifts in the time of initiation (heterochrony) contrast with a global proportional scaling of the process. An extreme example of heterochronies between fore- and hindlimbs are the marsupials. The earlier forelimb specification allows the neonate to climb to the pouch to complete development. Markers of forelimb are expressed relatively early in marsupials compared to other species, indicating that the forelimb fields arise earlier than in other mammals [50,51]. Altogether, heterochronies in limb indicate that these are more likely to occur at late stages.

## 7. Corticogenesis

The neocortex is the outer layer of the cerebral hemispheres and is unique to mammals. The neocortex has undergone substantial morphological transformations during evolution and shows a high degree of variation across mammals. It comprises six distinct layers of neurons that display specific patterns of gene expression and connectivity [52]. The layered organization of neurons is a consequence of the sequential differentiation of each neuronal subtype, with deeper layer neurons being generated earlier than upper layer neurons.

In contrast with the spinal cord, corticogenesis occurs over a protracted period in development, starting at mid-gestation stages and lasting until around birth in the mouse [52]. Recent studies have compared systematically and quantitatively the composition of the cortex *in vivo* and in stem cell models/organoids of corticogenesis across species [53–58]. These show that the phase of progenitor generation is extended in primates compared to rodents. Human progenitors proliferate for an extended period of time thus increasing the number of neurons [59]. In addition, the initiation of the neurogenic phase is delayed and extended in human versus mouse. This suggests that heterochronies, disproportionate changes in the timing of events, account for the evolutionary enlargement of the neocortex. It will be interesting to measure protein and mRNA kinetics in cortical progenitors to test if the mechanisms that correlate with the allochrony at the phylotypic stage are altered in this system.

Differences in tempo can also be measured independently of gene expression. A recent study comparing structural and functional maturation of neurons across primates shows that human-induced neurons develop slower than chimpanzee and bonobo neurons in terms of their electrophysiology and dendritic arborization [60]. Similarly, the period in which differentiating cortical neurons remain plastic is species-specific and associated with changes in mitochondria. This phase lasts 3 h post-mitosis in mouse and 6 h in human [61].

**Figure 2.**
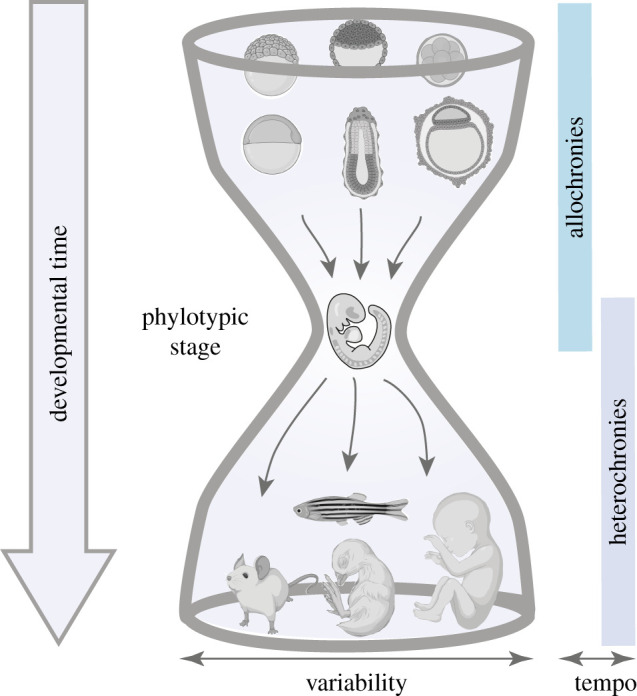
Hourglass model and tempo. The hourglass model proposes that mid-embryonic stages (phylotypic period) in vertebrate development represent the period of highest conservation, and that early and late developmental stages are highly variable. We propose that proportional temporal scaling (allochrony) is prevalent from the early to mid-stages of development. At late stages, there are frequent changes in the onset and/or duration of developmental processes (heterochronies). Embryos adapted from icons by BioRender.com.

Taken together, the available evidence suggests that there are proportional scaled changes in the pace of development (allochrony) during cleavage and the phylotypic stages, and that regulatory divergence observed in early stages maintains the allochrony of the system. By contrast, species-specific differences arising from other forms of heterochronies emerge at late stages of development and contribute to morphological variation (figure 2). Studying tempo in divergent developmental processes might help us define underlying evolutionary principles in development. The comparison between allochronic and other heterochronic processes will provide insight into the mechanistic and functional relevance during development.

## 8. Stopping the clock

Diapause is the delay in development initiated in response to adverse environmental conditions. It is a widespread physiological process in the animal kingdom where the developmental clock is stopped and then restarted [62]. How this is achieved remains poorly understood. The African turquoise killifish (*Nothobranchius furzeri*) uses diapause to survive long droughts. Killifish diapause embryos contain differentiated tissues from the three embryonic germ layers. It has been shown that the survival of the embryos preserves the organism for extremely long periods without trade-offs for adult growth, fertility and lifespan [63]. Diapause in killifish is maintained by specific chromatin regulators (the member of the Polycomb complex CBX7), and diapause embryos share a metabolic and chromatin signature with models of longevity in the worm *C. elegans* [63], suggesting a possible relationship between tempo control, epigenetics and longevity that we will later on refer to.

Some mammals also undergo diapause at the blastocyst stage, prior to gastrulation. This can be initiated as a response to cues indicating an adverse environment with development being resumed once environmental conditions improve [62]. In mouse, diapause can last up to two weeks, and it can be recapitulated *in vitro* [64,65]. This provides a tractable system for *in vivo* versus *in vitro* comparisons. Partial inhibition of mTOR, which is regulated by amino acid levels, induces reversible pausing of mouse blastocyst development [64,66]. The genetic mechanisms that are used during diapause entry and exit have only recently started to be explored, and it is tempting to speculate that the mechanisms that operate in diapause entry and exit correspond to those that control the developmental pace. Diapause could be a powerful system to uncover physiological mechanisms to slow down or accelerate developmental tempo.

## 9. Cell-autonomous tempo and transplantation

Xenotransplants, in which cells are transplanted from one species to another, provide evidence that in many cases developmental tempo is set cell autonomously. A recent report examining tempo mechanisms showed that zebrafish retina xenotransplanted in medaka resulted in a temporal decoupling between the donor retina and the host, supporting a species-specific autonomous tempo in biological systems [67].

Given the differences in tempo across species, it is worth reviewing the available knowledge in transplantation experiments used for disease modelling, replacement therapy or to generate human organs in animals for transplantation purposes.

### 9.1. Hematopoietic stem cells

Hematopoietic stem cells (HSCs) have the capacity to restore the entire hematopoietic system upon transplantation into a suitable recipient. The grafting of both mouse and human HSCs into immunodeficient mice has enabled the study of HSCs. The grafting efficiencies of human cells in mice are relatively low compared to the efficiencies associated with mouse–mouse transplants. This could be related to the state or the type of transplanted HSCs [68]. However, it is notable that grafted mouse HSCs can be readily detectable 10 days after transplantation, whereas human HSCs are generally detected only four weeks post-transplantation [69]. Even though differences in mouse and human HSCs populations exist, these data suggest a species-specific tempo of adult HSC differentiation.

### 9.2. Cortical neurons

Despite the relevance for brain evolution and diseases, the development of human neurons and circuits remains poorly understood. This is in part due to the challenge of studying live human neurons in the context of a brain circuit. *In vitro* differentiation models to study cortical neuron development are technically challenging because of the long-term maintenance of functional neurons. Therefore, xenotranplantations of human neurons into the mouse brain provide a means to study neuronal maturation under physiological conditions [70]. Embryonic cells that are introduced into the adult brain can integrate into neural circuits and function [71,72]. Human cortical pyramidal neurons xenotransplanted as single cells into the mouse cortex develop more slowly than similarly transplanted mouse neurons [73,74], indicating that neurons retain the maturation schedule of the species of origin, similar to *in vitro* studies. Despite the differences in the speed of progression, human neurons make synaptic connections in the mouse brain and respond to sensory stimuli in a similar fashion to host neurons.

### 9.3. Human organs grown in animals

Growing human organs in animals has received recent attention for transplantation purposes. One way this is achieved is by interspecies blastocyst complementation, where human pluripotent stem cells are injected into embryos from other species to generate chimeras. A puzzling question in these experiments has been the varying degrees of chimerism success [75]. It is assumed that mouse–rat chimeras develop at a high efficiency due to their relatedness. For instance, mouse chimeras with rat pancreas have been successfully generated by genetically modifying the mice so that their own cells could not develop into a pancreas [76]. By contrast, human stem cell engraftment in pig pre-implantation blastocysts show limited contribution at post-implantation stages, with only one human cell per 100 000 found in the host [77]. When monkey (*Macaca fascicularis*) stem cells are injected into pig blastocysts, only one in 1000–10 000 is from monkey origin [78]. One possibility, taking into account the differences in developmental progression between the species, is that slow-progressing cells are outcompeted by a faster developing host. In the future, matching the developmental progression of the host organism to generate human organs in animals will be key to overcome the species barrier.

## 10. Homeostasis and tempo

Organ homeostasis and regeneration in tissues such as skin, muscle, gut, liver, lungs, pancreas or blood depends on the balance between the proliferation and differentiation of tissue-resident stem cells in response to the environment, imbalances leading to ageing and disease. Controlling the dynamics of these processes is therefore essential to maintain functional organs in adult animals. As stem cell proliferation and differentiation are common mechanisms in the developmental and homeostatic process, differences in the speed of progression might exist across species during adult homeostasis.

The identification of mechanisms governing the regeneration and homeostasis in tissues has allowed the production of these tissues in three-dimensional *in vitro* culture systems, generally referred to as organoids. Organoids have been successfully obtained from mouse and human stomach, intestine, colon, lungs, pancreas, kidney and liver. Gastrointestinal organoids retain their self-renewing and differentiation potential, and their spatial organization recapitulates the *in vivo* organization of the epithelial intestine. Stem cells populate the base of bud-like structures and mature cell types migrate to the central structure of the cyst [61]. The speed at which cells in these organoids differentiate appears to be species-specific. For example, the generation of specialized goblet cells in intestinal organoids from stem cells in mouse cells takes approximately 2 days, whereas human goblet cells emerge around day 5 [79]. It would be interesting to systematically measure the tempo in the differentiation trajectories in comparable organoid models of mouse and human.

Similar to developmental organoids, these *in vitro* systems offer an unprecedented opportunity to investigate cell-autonomous and coordinated mechanisms responsible for the speed of progression. Whether the differences in developmental tempo between species reflects differences in the homeostatic processes that maintain mature organs in adult animals remains to be determined. The ability to understand the pace of progression during homeostasis may in turn allow us to speed up or slow down stem cells and optimize organoids for therapeutic applications and drug discovery. In addition, it might also provide insight relevant for slowing tumour growth.

## 11. Lifespan and tempo

**Figure 3.**
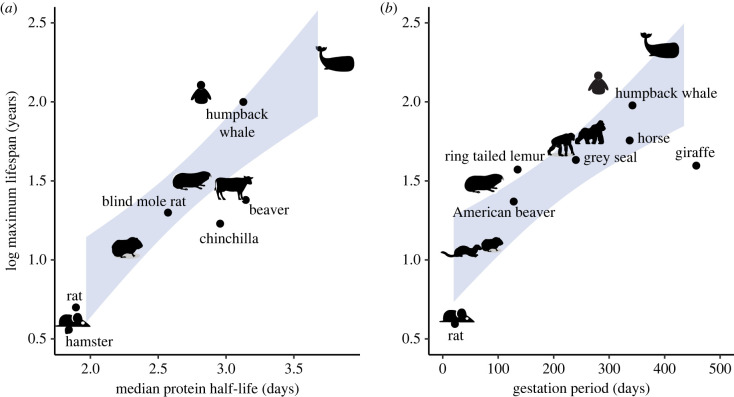
Lifespan, protein turnover and tempo. (*a*) Correlation between the gestation period and maximal lifespan across mammalian species. Data obtained from AnAge (https://genomics.senescence.info/species/). (*b*) Correlation between the species' median protein half-life values in senescent fibroblasts and maximal lifespans. Data taken from [83]. The shaded blue area spans the 95% confidence interval of a linear regressed model.

The rate of ageing is also species-specific and results in the characteristic average lifespans for a species [80,81]. Curiously, species with the longest lifespan tend to have longer gestational periods and are larger in size (figure 3*a*), although there are notable exceptions. For example, bats live longer than expected for their body size. A relationship between developmental progression and ageing has also been found in studies of the epigenetic clock. Temporal epigenetic changes in developmental GRNs are sufficient to estimate an epigenetic clock for human, dog and mouse [82].

Moreover, recent research demonstrates that organisms with long lifespans generally have slower global protein turnover rates [80,83] (figure 3*b*). These findings are in agreement with the identification of protein stability as a regulator of developmental tempo and suggest that the rate of protein turnover tracks the course of time from fertilization to death. It would be interesting to test if perturbations that extend the lifespan (caloric restriction, metformin treatment, etc.) also slow developmental tempo. Understanding the mechanisms behind developmental tempo may shed light on the molecular mechanisms behind size and lifespan scaling.

Highly abundant proteins in particular are more stable in long-lived animals [83]. The increased stability of highly abundant proteins minimizes the energetic expenditure (ATP demands) for the same biological process. Given that long-lived animals tend to be larger, the energetic cost of protein turnover in animals could be the molecular mechanism that determines the scaling between basal metabolic rate and organismal size (Kleiber's law). In the future, it will be key to identify the role that basal metabolic rate plays in protein turnover and developmental tempo.

## 12. Conclusion

Biological clocks can be identified during development and homeostasis in processes that are evolutionarily conserved across species. Cell-autonomous mechanisms are starting to be deciphered thanks to the conservation of GRNs and the use of *in vitro* models of development. Studying tempo in developmental processes, aside from the phylotypic stage, and during tissue homeostasis, will address whether there are general mechanisms that track time during development and homeostasis and how changes in tempo are achieved.

Moving beyond timers in single cells, three-dimensional models of development and homeostasis offer the potential to study the coordination of tempo at a tissue level. The development of comparable and robust protocols across species of organoids, and accurate dynamic measurements will be essential to gain insight into the dynamics of biological processes.

Moreover, the identification of tempo mechanisms will allow us to investigate how to modulate the speed of progression. This will not only improve our understanding of biological processes but is likely to provide the means to engineer and refine methods to generate specific cell types for research and therapeutic applications.

## Data accessibility

Data for figure 3 are available from AnAge (https://genomics.senescence.info/species/) and electronic supplementary material from [83].
